# Are NCCN Resource-Stratified Guidelines for Breast Cancer Systemic Therapy Achievable? A Population-Based Study of Global Need and Economic Impact

**DOI:** 10.1200/GO.21.00028

**Published:** 2021-07-06

**Authors:** Brooke E. Wilson, Susannah Jacob, Viet Do, Eitan Amir, Freddie Bray, Jacques Ferlay, Felicia M. Knaul, Ahmed Elawawy, Sallie-Anne Pearson, Michael B. Barton

**Affiliations:** ^1^ Collaboration for Cancer Outcomes, Research and Evaluation, South West Clinical School, University of New South Wales, Liverpool, New South Wales, Australia; ^2^ Princess Margaret Hospital, University of Toronto, Toronto, Ontario, Canada; ^3^ Liverpool Hospital, Department of Radiation Oncology, Liverpool, New South Wales, Australia; ^4^ Cancer Surveillance Section, International Agency for Cancer Research, Lyon, France; ^5^ Sylvester Comprehensive Cancer Center, University of Miami, Miami, FL; ^6^ Department of Public Health Sciences, Leonard M. Miller School of Medicine, Miami, FL; ^7^ Institute for Advanced Study of the Americas, University of Miami, Coral Gables, FL; ^8^ Tómatelo a Pecho, A.C., Mexico City, Mexico; ^9^ Mexican Health Foundation (FUNSALUD), Mexico City, Mexico; ^10^ Suez Canal University, Ismailia, Egypt; ^11^ Alsoliman Radiation and Oncology Center, Port Said, Egypt; ^12^ Centre for Big Data Research in Health, UNSW, Sydney, Australia; ^13^ Menzies Centre for Health Policy, University of Sydney, Sydney, Australia

## Abstract

Resource-stratified guidelines (RSG) for cancer provide a hierarchy of interventions, based on resource availability. We quantify treatment need and cost if National Comprehensive Cancer Network (NCCN) RSGs for breast cancer (BC) are adopted globally.

**METHODS:**

We developed decision trees for first-course systemic therapy, merged with SEER and Global Cancer Observatory 2018 incidence data to estimate treatment need and cost if NCCN RSG are implemented globally based on country-level income. Simulations were used to quantify need and cost of globally scaling up services to Maximal.

**RESULTS:**

Based on NCCN RSG, first-course chemotherapy is indicated in 0% (Basic), 87% (Core), and 86% (Enhanced) but declined to 50% (Maximal) because of incorporation of genomic profiling. First-course endocrine therapy (ET) is indicated in 80% in all settings. In 2018, treatment need was 1.4 million people for chemotherapy, 183,943 for human epidermal growth factor receptor 2 (HER2) therapies and 1.6 million for ET. The cost per person for chemotherapy or HER2 or immunotherapy increased by 17-fold from Core to Maximal ($1,278-$22,313 Australian dollars [AUD]). The cost of ET per person rose eight-fold from Basic to Maximal ($1,236-$9,809 AUD). If all patients with BC globally were treated with Maximal resources, the need for chemotherapy would decline by 28%, whereas cost of first-course treatment would rise by 1.8-fold ($21-$37 billion AUD) because of more costly therapies.

**CONCLUSION:**

NCCN RSGs for BC could result in chemotherapy overtreatment in Core and Enhanced settings. The absence of chemotherapy in Basic settings should be reconsidered, and future iterations of RSG should perform cross-tumor comparisons to ensure equitable resource distribution and maximize population-level outcomes. Our model is flexible and can be tailored to the costs, population attributes, and resource availability of any institution or country for health-services planning.

## INTRODUCTION

Breast cancer (BC) accounts for 11.6% of new cancers and 6.6% of all cancer-related deaths.^1^ If all countries delivered chemotherapy based on best-practice guidelines in 2018, approximately 1.4 million people would have benefitted from BC chemotherapy.^2^ Low- and middle-income countries (LMICs) account for approximately 60% of global cases^1^ and have higher mortality-to-incidence ratios than high-income countries.^1,3^ As LMICs continue to undergo economic and demographic transition, rising treatment needs will place increasing demands on largely underprepared health systems.

**CONTEXT**

**Key Objective**
To estimate the need and cost of systemic breast cancer (BC) treatment if delivered globally according to resource-stratified guidelines, and to model the upscaling of service provision toward maximal resources for all patients regardless of income group.
**Knowledge Generated**
If National Comprehensive Cancer Network resource-stratified guidelines for BC are adopted globally based on country-level income, the need for chemotherapy would be 1.4 million persons per year. Delivering resource-stratified guidelines–based care risks overtreating patients in Core (low-middle income) and Enhanced (upper-middle income) settings and undertreated those in the Basic (low-income) setting.
**Relevance**
Our findings, and the flexible model presented herein, will inform health-services planning for both high-income countries and low- and middle-income countries. These results can also inform future iterations of resource-stratified guidelines for BC treatment.

The National Comprehensive Cancer Network (NCCN) has developed resource-stratified guidelines (RSG) for BC, identifying a hierarchy of interventions based on level of economic development. The hierarchy considers the contribution of each treatment to overall survival, disease-free survival, quality of life, and cost.^4^ Treatment levels are Basic,^5^ Core,^6^ Enhanced,^7^ and Maximal,^8^ based on earlier guidelines from the Breast Health Global Initiative.^9^ Although they offer a transparent and plausible approach to guiding treatment decisions in different resource settings, no studies have examined the economic impact or treatment needs if RSGs are widely adopted.

We estimated the resource-stratified need and economic impact of first-course systemic BC treatment (including chemotherapy, endocrine therapy, and targeted therapy) if delivered globally according to NCCN RSG by level of economic development. We also estimated the change in need and cost if service provision for systemic therapy is scaled up globally toward Maximal. We also developed a flexible user interface to tailor the estimates of cost and need using local input data.

## METHODS

### Decision Tree

We developed treatment decision trees for first-line chemotherapy, immunotherapy, endocrine therapy (ET), cyclin-dependent kinase 4/6 inhibitors, and human epidermal growth factor receptor 2 (HER2) therapy using TreeAgePro2018^10^ for Basic,^5^ Core,^6^ Enhanced,^7^ and Maximal^8^ services outlined in the NCCN RSG. Basic and Core guidelines are based on stage, hormone receptor, and performance status, whereas Enhanced incorporates HER2, and Maximal includes multigene signatures and programmed death ligand-1 expression. We considered only first-course treatment (either adjuvant or metastatic) for each modality. We define need as medically indicated by the NCCN RSG.

NCCN recommendations are category-based, relating to the strength of evidence and consensus (Data Supplement). We modeled category 1 recommendations for adjuvant chemotherapy and HER2 therapies, category 2A for metastatic chemotherapy, and category 1, 2A, and 2B for ET.

To determine the proportion of patients in each decision branch, we extracted data from SEER,^11^ or best available evidence in the absence of SEER data (Data Supplement). Because of a paucity of data in LMICs, we assume that histologic and patient characteristics and stage of distribution are the same in all income settings. The implications of a constant stage distribution are described and modeled elsewhere.^2^

Each country was assigned a resource group based on World Bank Development Classification 2018^12^ income levels (Low-income [L]—Basic; Low-middle [LM]—Core; Upper-middle [UM]—Enhanced; High-income [H]—Maximal). We extracted BC incidence by country in 2018 and projected to 2040 based on linear population growth from Global Cancer Observatory (GLOBOCAN).^1^ We then multiplied each country-level incidence by the appropriate income-based RSG tree to estimate the need for treatment by country. We then pooled all country-level estimates to provide global need estimates.

### Preferred Treatment Regimens

We extracted the preferred first-line chemotherapy, immunotherapy, HER2, and ET for each setting and resource level from NCCN RSG (Data Supplement). We assumed that single-agent chemotherapy for metastatic disease is preferred,^13^ and that recommended adjuvant and metastatic regimens are used with equal probability. We excluded NCCN recommended treatments not commonly used as a first-line therapy. We assume that if used, gonadotropin-releasing hormone agonists are given for 5 years. We assume that the available treatment options do not change over time. A complete list of model assumptions is found in the Data Supplement.

### Model Costs

From the Australian Pharmaceutical Benefits Scheme (PBS), we extracted the lowest listed cost per unit from January 2020,^14^ in Australian Dollars. We compared the PBS cost with the International Medical Products Price Guide 2015 from the Management Sciences for Health (MSH)^15^ where possible (Data Supplement). As cost varies depending on country and income level,^15,16^ we focus on relative cost differences rather than absolute costs. We assumed fixed costs between 2018 and 2040.

Dose calculations were based on a body surface area of 1.8 m^2^, weight of 75 kg, and creatinine clearance of 75 mL/min. Growth factor support, gene expression test costs (Oncotype Dx), and nondrug prices (programmed death ligand-1 testing and oophorectomy) can be found in the Data Supplement. Where a range of treatment durations for adjuvant ET are possible, we used the mean to derive the regimen cost. In the metastatic setting, we estimated cost from the median time to progression or progression-free survival in the pivotal trials referenced in the NCCN RSG. The costs of drug delivery and hospitalizations were not included.

### Sensitivity Analysis

We performed three sensitivity analyses. We examined the change in chemotherapy need for ER-positive, HER2-negative, node-negative BCs, based on prior model estimates incorporating additional clinical and histologic features.^17^ We explored the impact of country-level price differences using the lowest and the highest priced regimen, and half the lowest and twice the highest priced regimen for each indication. Finally, we generated seven scenarios, where the resource level assigned to each income level is progressively increased from Basic toward Maximal to model the upscaling of services globally.

## RESULTS

Our model resulted in a decision tree with more than 300 independent decision nodes used to determine treatment for any given patient (Data Supplement).

### Global BC Incidence by Income Group

Based on GLOBOCAN 2018,^1^ the proportion of BC cases by income group in 2018 was 3% in L (70,401/2,072,659), 21% in LM (442,075), 36% in UM (736,657), and 40% (823,526) in H income countries. Based on population projections, the number of BC cases will rise to 2,798,001 by 2040, with an increasing proportion occurring in LMICs (5% in L, 26% in LM, 34% in UM, and 35% in H) (Table 1).

**TABLE 1:**
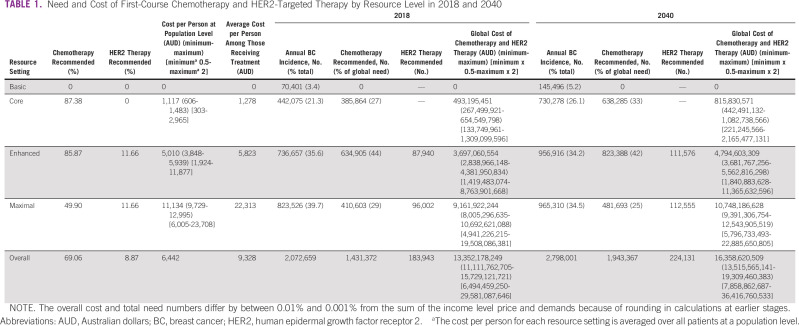
Need and Cost of First-Course Chemotherapy and HER2-Targeted Therapy by Resource Level in 2018 and 2040

### Resource-Stratified Need for Chemotherapy

Chemotherapy recommendations differ across resource levels (Data Supplement). Basic guidelines do not recommend chemotherapy for any patients. Applying Core and Enhanced guidelines, 87% and 86% of patients, respectively, have an indication for at least one line of chemotherapy, driven by the category 1 recommendation for chemotherapy for most patients with node-negative ER-positive disease. By incorporating additional variables such as grade, age, and patient preference,^17^ the need for chemotherapy falls to 70% (Core) and 75% (Enhanced). In the Maximal setting, chemotherapy need falls to 50% after incorporating multigene signatures (Table 1).

Merging these RSG treatment rates with BC incidence, we estimate that 69% of patients globally have an indication for first-course chemotherapy in 2018 (1,431,372/2,072,659). None reside in L, 385,864 (27%) in LM, 634,905 (44%) in UM, and 410,603 (29%) in H income countries. By 2040, need for first-course chemotherapy will rise to 1,943,367 (638,285 [33%] in LM; 823,388 [42%] in UM; 481,693 [25%] in H) (Table 1).

### HER2 and Immunotherapy Need

HER2 therapies are recommended in 12% of patients in Enhanced and Maximal, and none in Basic and Core settings. Merging these data with GLOBOCAN cancer incidence, almost 9% of all patients with BC in 2018 have an indication for HER2 therapies, but only 8% in 2040 because of rising incidence in L and LM countries. The absolute number of patients recommended HER2 therapies increases from 183,943 to 224,131 between 2018 and 2040 because of population growth (Table 1). First-course immunotherapy for metastatic triple-negative BC is only recommended in the Maximal setting and is indicated in 0.2% of patients in 2018 (3,946 persons).

### Costs of First-Course Chemotherapy, HER2 Therapy, and Immunotherapy Treatments

The cost of guideline-recommended, first-course chemotherapy in LM applying Core resources is $1,278 US dollars (USD) per person (pp) needing treatment, whereas the average cost of chemotherapy and HER2 therapy in UM applying Enhanced guidelines is $5,823 AUD pp needing treatment (Table 1, Data Supplement). The rise in cost between Core and Enhanced is driven predominantly by the incorporation of HER2 therapies. For H applying Maximal guidelines, the average cost is $22,313 AUD pp needing treatment, driven by the incorporation of dose-dense chemotherapies requiring growth factor support, HER2 therapies, and immunotherapy (Table 1, Data Supplement).

**FIG 1:**
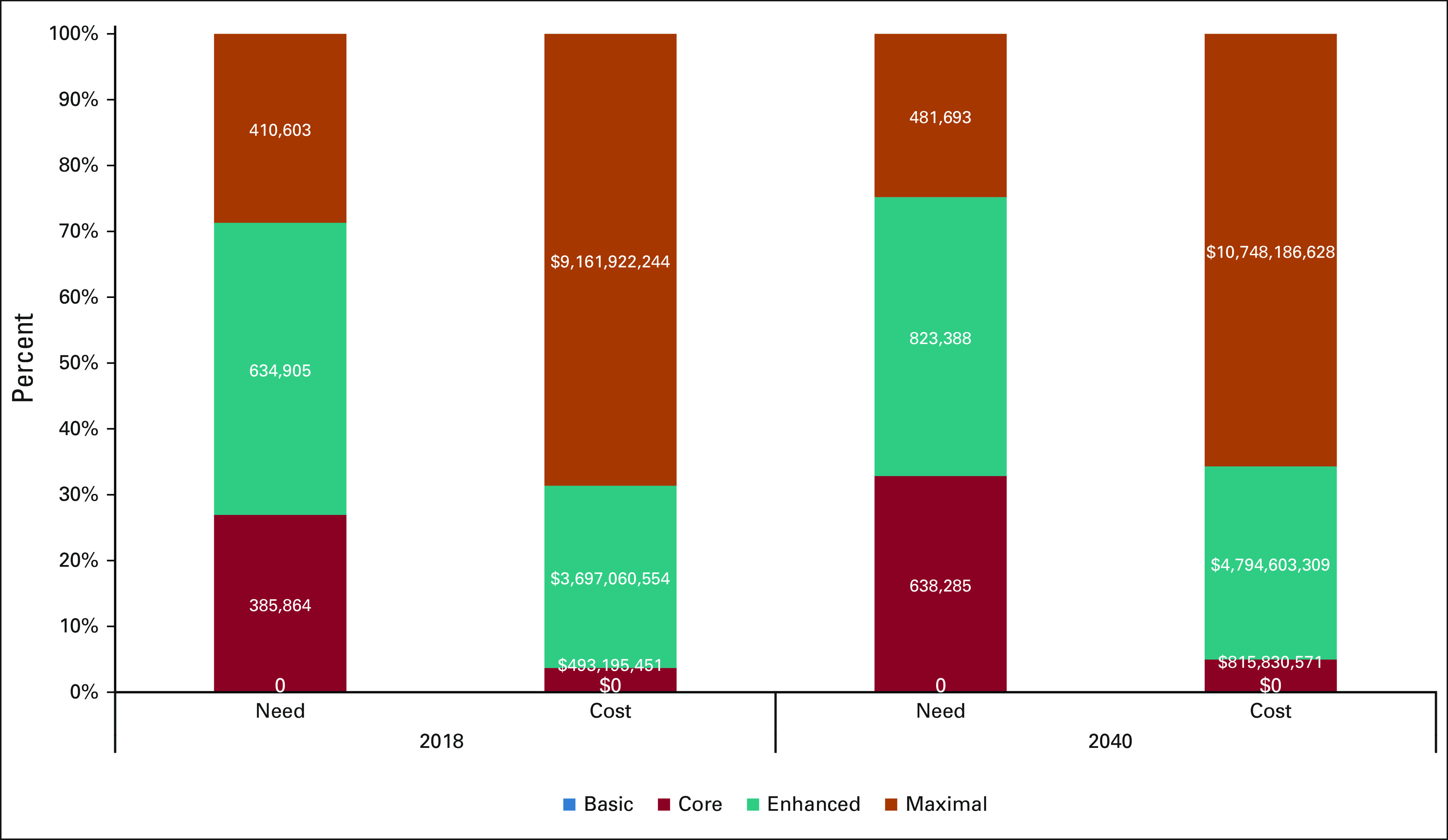
Proportion of global chemotherapy or HER2 cost and need by income group in 2018 and 2040. The estimated need for chemotherapy and cost for chemotherapy or HER2 therapy by resource level (Basic, Core, Enhanced, and Maximal) as a proportion of total global demand in 2018 and 2040, if treatments are delivered globally based on National Comprehensive Cancer Network resource-stratified guidelines. HER2, human epidermal growth factor receptor 2.

As absolute costs (Table 1) are subject to country-level pricing, we report relative cost changes from service expansion. The relative cost pp for first-course chemotherapy, HER2 therapy, and immunotherapy as we move from Core to Maximal resources increases by 17-fold (from $1,278 to $22,313 AUD). The economic cost of adopting first-course NCCN BC RSG globally is $13.3 billion AUD in 2018 and rises to $16.3 billion AUD in 2040. Although LMICS will comprise 75% of global need for chemotherapy and HER2 therapy by 2040 if NCCN RSG are widely adopted, according to our projections, they will account for only 34% of global expenditures (Fig 1). Sensitivity analyses of drug cost provide a global range of $7.8-$36.4 AUD billion in 2040 (Table 1).

### Endocrine Therapy Need and Costs

Although the need for first-course ET based on NCCN RSG is 80% in all settings (Table 2), the types and cost of recommended therapies vary by resource level (Data Supplement). First-course ET is indicated in 1,658,038 people in 2018 and 2,238,272 in 2040 because of population growth.

**TABLE 2:**
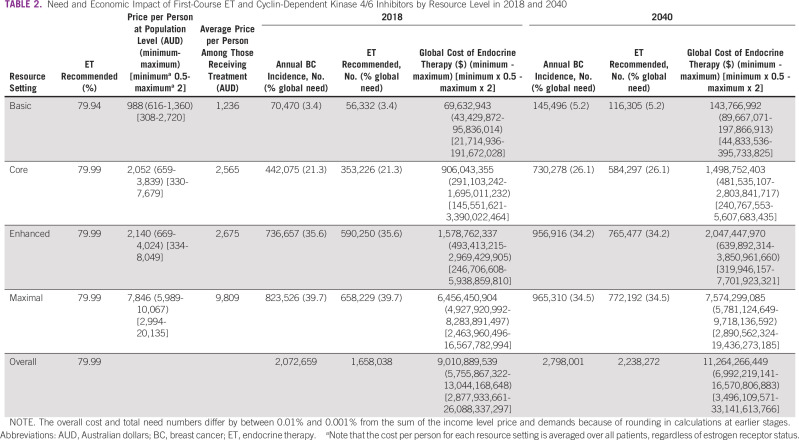
Need and Economic Impact of First-Course ET and Cyclin-Dependent Kinase 4/6 Inhibitors by Resource Level in 2018 and 2040

**FIG 2:**
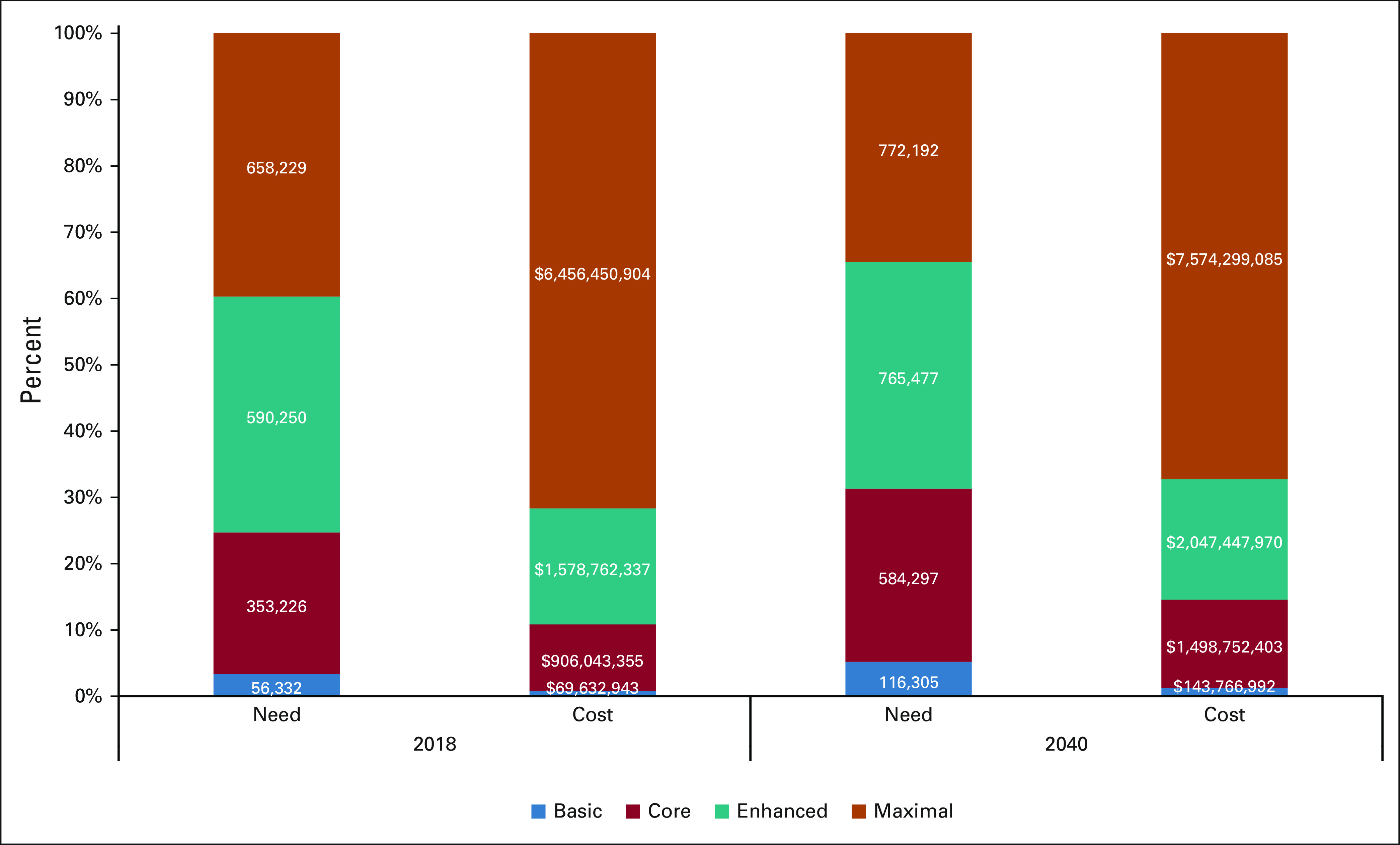
Proportion of global ET cost and need by income group in 2018 and 2040. The estimated cost and need for ET by resource level (Basic, Core, Enhanced, and Maximal) as a proportion of total global demand in 2018 and 2040, if treatments are delivered globally based on National Comprehensive Cancer Network resource-stratified guidelines. ET, endocrine therapy.

The average cost of ET in the Basic setting is $1,236 AUD pp needing treatment, where only tamoxifen and oophorectomy are recommended. The cost of ET is eight-fold higher in the Maximal resource setting ($9,809 AUD pp needing treatment), driven by the incorporation of gonadotropin-releasing hormone agonists and cyclin-dependent kinase 4/6 inhibitors (Table 2, Data Supplement). The estimated cost of globally adopting NCCN RSG for ET is $9 billion AUD in 2018 and $11.3 billion AUD in 2040 (Table 2). Sensitivity analyses of global cost provide a range of $3.5-$33.1 billion AUD in 2040 (Table 2). Whereas 66% of global need for ET will be in LMICs by 2040, only 33% of global expenditures will be in LMICs if NCCN BC RSG are widely adopted (Fig 2).

### Upscaling Service Provision Globally to Maximal

**FIG 3:**
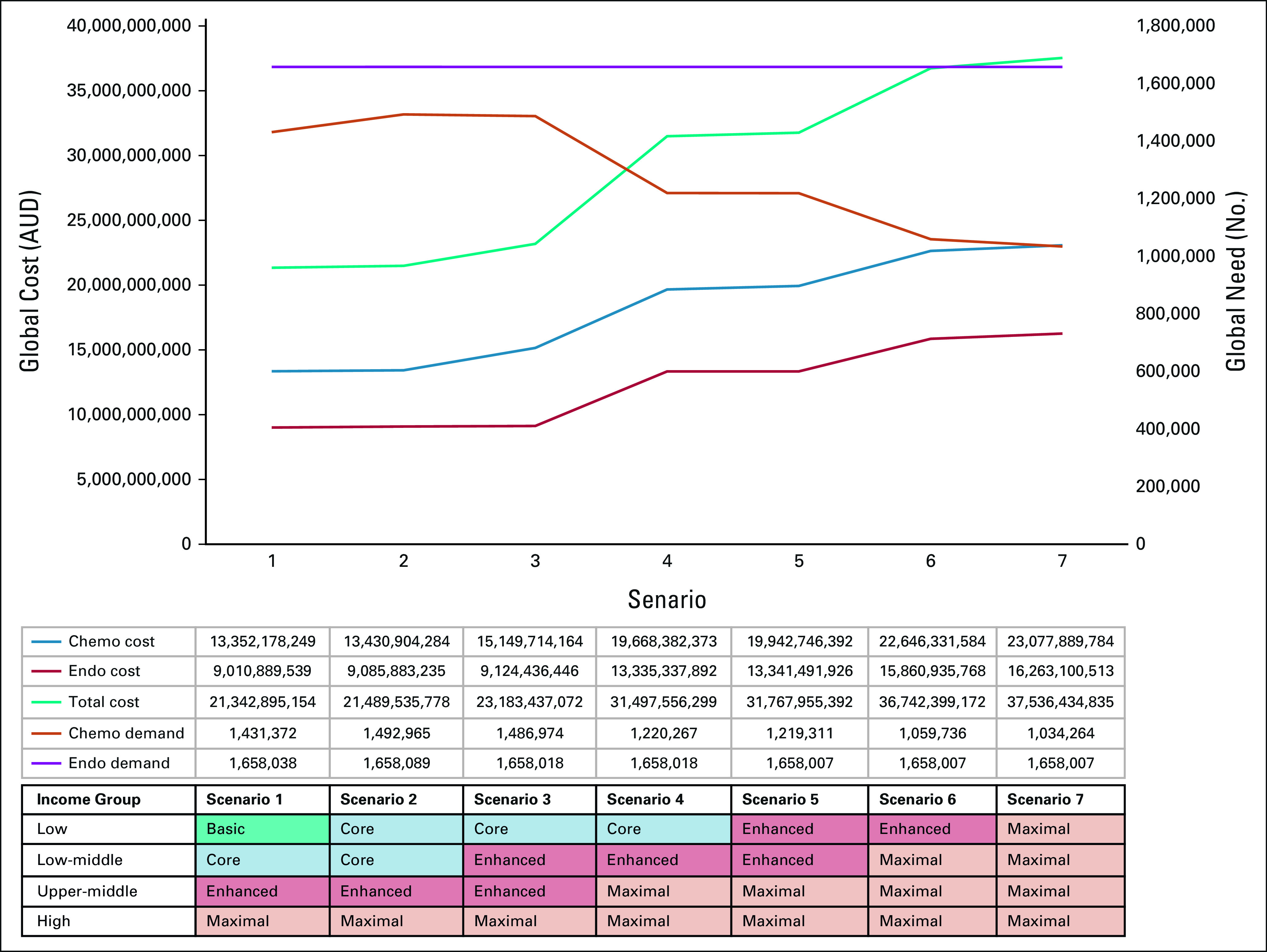
Global need and economic impact of first-course chemotherapy or HER2 and endocrine treatment as service provision is upscaled globally in 2018. This figure depicts the global treatment need for endocrine therapy and chemotherapy (including HER2 treatment and immunotherapy) and the cost for endocrine and chemotherapy or HER2 treatments as service provision is upscaled globally toward Maximal based on National Comprehensive Cancer Network resource-stratified guidelines for breast cancer. Scenarios 1-7 depict increasing resource availability for each income group, from the base scenario (scenario 1) to the maximal scenario where all patients are treated with maximal resource availability (scenario 7), regardless of income group. AUD, Australian dollars; chemo, chemotherapy; endo, endocrine therapy; HER2, human epidermal growth factor receptor 2.

As service provision is upscaled toward Maximal in all countries, we found paradoxically a 28% decline in guideline-based chemotherapy need (from 1,431,372 to 1,034,264) (Fig 3). This is driven by incorporating multigene signatures in Maximal settings, allowing for better selection of patients with node-negative, ER-positive disease. Despite this reduction in need, the global chemotherapy, HER2 therapy, and immunotherapy cost rises 1.7-fold (from $13 to $23 billion AUD), whereas ET increases 1.8-fold (from $9 to $16 billion AUD), because of more costly treatments (Fig 3). The total cost of guideline-based first-course treatment for BC in 2018 rises 1.8-fold (from $22 to $39 billion AUD) if all people with BC are treated with Maximal resources.

### Tailoring Model Inputs

In generating the current model, we made assumptions regarding population attributes, drug costs, stage distribution, and global resource availability (Data Supplement). However, this model is flexible, and an adaptable user interface is provided in the Data Supplement. The input parameters can be adjusted to the specific population, cost, and resource characteristics of any institution, region, or country to estimate need and cost for first-course treatment of BC for health-service planning (Data Supplement).

## DISCUSSION

Based on NCCN RSG, the global resource-stratified need for BC chemotherapy in 2018 is 1.4 million people, the need for HER2 treatments is 183,943 people, and the need for first-course ET is 1.6 million people. If all patients are treated according to Maximal guidelines, relative global expenditure for systemic BC treatment would increase by 1.8-fold, whereas chemotherapy need would decline by 28% because of better selection of patients most likely to benefit. ET need would remain unchanged. This is a relatively modest increase especially if offset by the decrease in costs of toxicity that was not included in our model.

Overtreatment is costly to patients and health systems. If the optimal proportion of patients with BC needing chemotherapy is 50% as calculated in the Maximal setting, adopting NCCN RSG could result in overtreatment of up to 37% (87%-50%) and 36% (86%-50%) of patients in Core and Enhanced settings, respectively, because of the category 1 recommendation for chemotherapy for patients with node-negative ER-positive cancers ≥ 0.5 cm. In reality, many of these patients would receive ET alone (category 2A recommendation), even in the absence of multigene signatures, based on clinician judgment. Sensitivity analysis demonstrates that using additional histologic and clinical variables to de-escalate chemotherapy could reduce need to 70.4% (Core) and 75.2% (Enhanced). Having clear guidelines that do not result in overtreatment for marginal gains, especially in limited-resource settings, is important to avoid toxicity and maximize population-level cost effectiveness.

The NCCN RSG categories of evidence are extrapolations of results from Maximal settings where most trials are conducted. Even within the constraints of clinical trials offering the same treatment and monitoring, outcomes are often worse in LMICs.^18^ With this in mind, it may be advantageous to reconsider the definitions applied for RSG category-based recommendations, prioritizing the value of a therapy in addition to the strength of evidence, and further incorporating trial and real-world data from LMICs.

The assumption that no chemotherapy is available in the Basic setting should be reconsidered. If we assume Core resources in all low-income countries, global chemotherapy need increases by 61,593 with an additional cost of $78 million AUD. The absence of chemotherapy in the Basic NCCN RSG for BC conflicts with Basic NCCN RSG for colon cancer where adjuvant and palliative chemotherapy are recommended.^19^ The evidence supporting adjuvant chemotherapy for high-risk BC is clear,^20,21^ with survival benefits equal to or greater than for adjuvant chemotherapy for colon cancer.^22^ The NCCN BC guidelines also conflict with the BGHI^9^ RSGs and Asian Oncology RSGs for HER2-positive disease,^23^ which both support chemotherapy in the Basic setting. Future iterations of RSGs should consider population-based incremental costs and benefits and perform cross-tumor comparisons and validations to ensure resources are allocated consistently between cancer types to achieve maximum, equitable health benefits.

Our paper focuses on quantifying the global need and cost of providing BC treatment based on NCCN RSG. In implementing these guidelines, there are numerous challenges such as the availability of imaging and pathology services for diagnosis, drug availability and funding for treatment, availability of surgical and radiation services, and the training of medical and nursing practitioners to deliver care. Much can be learned from the global upscaling of treatment for individuals with HIV, where the development of global training programs, low-cost reliable generic medication, and bulk purchasing led to significant improvements in access to care. As previously advocated, a similar approach to implementing cancer care globally should be considered, and could decrease the global costs estimated in this model.

Guidelines by definition are imperfect and represent only a reference document from which decisions can be made. It can be difficult to determine the best evidence as it applies to all clinical circumstances,^24^ and category 1 recommendations may not always be appropriate. Clinicians should use medical judgment and consider individual patient circumstances when determining care.^8^ Actual treatment decisions may be influenced by incentive payments, drug availability,^25,26^ or clinical determinants not captured by guidelines. These limitations of the NCCN guidelines should be considered when interpreting our results.

The NCCN RSG were developed to allow heterogeneity of service provision within countries. Our model assumes that the same level of care is provided across an entire country and that care levels correlate with a country's income. Even in high-income countries, significant heterogeneity in outcomes exists,^27^ which may in part reflect differences in access to screening and treatment. In LMICs such as India, substantial heterogeneity in treatment also exists, with some centers offering care at the standard of high-income countries, whereas many rural areas have limited services.^28,29^ Nonetheless, this study provides a benchmark for global need and economic impact if countries apply care models matched to their income group homogenously within their borders. The flexible user interface in the Data Supplement allows input parameters to be adapted to any individual country or region based on local patient characteristics, costs, and heterogeneity of resource availability.

A complete list of assumptions and their implications are given in the Data Supplement. A limitation of our economic analysis is the lack of comprehensive publicly available global pricing data. We explored differences in country-level cost through sensitivity analysis and compared cost to MSH listings where possible. Full model simulation using MSH prices was not possible as key therapies (eg, trastuzumab) were missing. Furthermore, MSH costs were often based on few datapoints and may not be representative of the true global market. The full cost of drugs to governments may not be reflected in the PBS listed price, and these estimates should be viewed as conservative. Moreover, this paper does not include the costs associated with imaging, surgery, pathology, radiation, palliative care, and nursing, which are all integral components of comprehensive BC care.

It is well recognized that women in LMICs present with more advanced disease, and therefore the current estimates for cost and need likely underestimate the true global utilization, as we have demonstrated in prior published models.^2^ Although we did not explicitly model potential differences in stage distribution between resource levels in this current paper, this can be done using the flexible user interface provided. Only the need and cost of first-course therapies were included in our model. Second and third courses of treatment are important on a global scale to gain a complete understanding of system costs. However, there are limited data to inform calculations on the prevalence of subsequent courses of treatment as they are often conditional on receipt of prior lines and changes to patient fitness over time. Moreover, the magnitude of survival benefits of second-line and third-line chemotherapy is often smaller and more variable.^30^ The lack of survival data associated with each level of service provision is another important limitation that is the subject of ongoing research.

In conclusion, based on our model, 1.4 million people need first-course BC chemotherapy, 183,943 HER2 therapy, and 1.6 million ET in 2018. NCCN RSG for BC could result in overtreatment of patients in Core and Enhanced settings. As global service provision is upscaled toward Maximal, chemotherapy need declines by 28%, whereas total cost rises by 1.8-fold (from $21 to $37 billion AUD). The absence of chemotherapy in Basic settings should be reconsidered to allow equitable resource allocation across tumor types. This model is flexible and can estimate the need and cost of implementing NCCN BC RSG for any system based on their unique resource mix, drug costs, and population attributes.
